# An Aboriginal and Torres Strait Islander Cardiac Rehabilitation program delivered in a non-Indigenous health service (Yeddung Gauar): a mixed methods feasibility study

**DOI:** 10.1186/s12872-021-02016-3

**Published:** 2021-05-01

**Authors:** Nicole Freene, Roslyn Brown, Paul Collis, Chris Bourke, Katharine Silk, Alicia Jackson, Rachel Davey, Holly L. Northam

**Affiliations:** 1 Physiotherapy, Faculty of Health, University of Canberra, Bruce, ACT 2617 Australia; 2 Health Research Institute, University of Canberra, Bruce, ACT Australia; 3 Ngunnawal Centre, Office of Aboriginal and Torres Strait Islander Leadership and Strategy, University of Canberra, Bruce, ACT Australia; 4 Faculty of Arts and Design, University of Canberra, Bruce, ACT Australia; 5 Australian Healthcare and Hospitals Association, Deakin, ACT Australia; 6 Nursing, Midwifery and Public Health, Faculty of Health, University of Canberra, Bruce, ACT Australia

**Keywords:** Indigenous health, Cardiovascular disease, Coronary heart disease, Prevention, Health workforce, Cultural safety

## Abstract

**Background:**

There is limited evidence of Aboriginal and Torres Strait Islander people attending cardiac rehabilitation (CR) programs despite high levels of heart disease. One key enabler for CR attendance is a culturally safe program. This study evaluates improving access for Aboriginal and Torres Strait Islander women to attend a CR program in a non-Indigenous health service, alongside improving health workforce cultural safety.

**Methods:**

An 18-week mixed-methods feasibility study was conducted, with weekly flexible CR sessions delivered by a multidisciplinary team and an Aboriginal and/or Torres Strait Islander Health Worker (AHW) at a university health centre. Aboriginal and Torres Strait Islander women who were at risk of, or had experienced, a cardiac event were recruited. Data was collected from participants at baseline, and at every sixth-session attended, including measures of disease risk, quality-of-life, exercise capacity and anxiety and depression. Cultural awareness training was provided for health professionals before the program commenced. Assessment of health professionals’ cultural awareness pre- and post-program was evaluated using a questionnaire (n = 18). Qualitative data from participants (n = 3), the AHW, health professionals (n = 4) and referrers (n = 4) was collected at the end of the program using yarning methodology and analysed thematically using Charmaz’s constant comparative approach.

**Results:**

Eight referrals were received for the CR program and four Aboriginal women attended the program, aged from 24 to 68 years. Adherence to the weekly sessions ranged from 65 to 100%. At the program’s conclusion, there was a significant change in health professionals’ perception of social policies implemented to ‘improve’ Aboriginal people, and self-reported changes in health professionals’ behaviours and skills. Themes were identified for recruitment, participants, health professionals and program delivery, with cultural safety enveloping all areas. Trust was a major theme for recruitment and adherence of participants. The AHW was a key enabler of cultural authenticity, and the flexibility of the program contributed greatly to participant perceptions of cultural safety. Barriers for attendance were not unique to this population.

**Conclusion:**

The flexible CR program in a non-Indigenous service provided a culturally safe environment for Aboriginal women but referrals were low. Importantly, the combination of cultural awareness training and participation in the program delivery improved health professionals’ confidence in working with Aboriginal people. *Trial registration*: Australian New Zealand Clinical Trials Registry (ANZCTR) 12618000581268, http://www.ANZCTR.org.au/ACTRN12618000581268.aspx, registered 16 April 2018.

**Supplementary Information:**

The online version contains supplementary material available at 10.1186/s12872-021-02016-3.

## Background

Cardiovascular disease (CVD) is diagnosed in almost half of Aboriginal and Torres Strait Islander people aged 55 years and over, and is the largest cause of premature death in this population [1]. CVD is the primary influencing factor in the life expectancy gap between Aboriginal and Torres Strait Islander people and non-Indigenous populations [1]. Ischaemic heart disease alone is reported to account for 24% of the avoidable and preventable gap, with Aboriginal and Torres Strait Islander people being three times more likely to have a major cardiac event [1–5]. In addition to this, Aboriginal and Torres Strait Islander people are more than twice as likely to die in hospital from heart disease, and are more likely to have numerous cardiac risk factors such as smoking, high blood pressure, obesity and diabetes [5, 6].

It is widely recognised that cardiac rehabilitation (CR) improves cardio-metabolic risk profiles, decreases hospital admissions, increases medication adherence and improves quality-of-life in those diagnosed with coronary heart disease [7–9]. Cardiac rehabilitation includes exercise, education and psychosocial components, and in Australia, the term CR is often used to refer to structured, short-term, centre-based, out-patient programs [10, 11]. However, there are many barriers to participation for Aboriginal and Torres Strait Islander people [10]. Studies have demonstrated that the main barriers to CR attendance are inadequate access to care due to geographical location, patient misconceptions of the purposes and benefits of CR, sub-optimal awareness by practitioners of the national guidelines for the implementation of CR, and inadequate patient–physician communication [12–16]. Barriers reported by Aboriginal and Torres Strait Islander people with cardiac disease include extended family responsibilities, sociocultural inappropriateness of the program, the connection between colonialism and health services, negative media messages regarding Aboriginal and Torres Strait Islander heart health resulting in disempowerment, and the younger age at which Aboriginal and Torres Strait Islander people are diagnosed, making them feel uncomfortable when attending a group with older participants [13].

Despite these barriers, CR programs have been implemented for Aboriginal and Torres Strait Islander populations, primarily in Aboriginal Medical Services (AMS) [17, 18]. These programs have resulted in significant decreases in waist circumference and blood pressure, and increases in exercise capacity and quality-of-life [17, 18]. Strategies shown to be successful to increase participation in CR for this population are the inclusion of Aboriginal and/or Torres Strait Islander Health Workers (AHW), provision of transport assistance and education in gender specific, culturally sensitive, structured and flexible CR programs [16–19]. Yet, participation of Aboriginal and Torres Strait Islander people in CR remains very low [10, 15, 20, 21], with some indication only 3–5% of those eligible attend [22] compared with 9% in non-Indigenous populations [23], however data is limited.

One of the key enablers for Aboriginal and Torres Strait Islander CR attendance is the provision of a culturally safe program, preferably delivered within an AMS [18, 19]. Cultural safety was developed in New Zealand to address the ways colonial processes and structure negatively affect Maori health [24]. It is defined by recipients of the care or service, and emphasizes the important role empathy and reflective practice play when used by health professionals to reduce patients alienation from healthcare services, prioritizing patient-centred care [25, 26]. It is recognised that CR for Aboriginal and Torres Islander people is best delivered in an AMS to ensure cultural safety. However, a local AMS is not always available in areas of need, with over 25% of Indigenous Australians reporting that they did not have an AMS within their local area [2]. Therefore, non-AMS health services must provide culturally safe care and improvements must be made to our existing cardiovascular care workforce to reduce the gap in life expectancy [15, 21, 27]. Improving the cultural safety of CR programs can increase utilisation of CR services for Aboriginal and Torres Strait Islander people, recognising the historical, social and cultural circumstances of Indigenous Australians and how these act directly as a barrier to health service engagement [21, 28, 29]. A multi-strategy approach to improve access and outcomes in healthcare for Aboriginal and Torres Strait Islander people is recommended, although the best combination of strategies to improve access and outcomes in healthcare for Aboriginal and Torres Strait Islander people is unknown [24].

The aim of this study was to implement known enablers, including the provision of a culturally informed gender-specific program, to overcome common access barriers for participation in CR programs in a non-Indigenous health service for Aboriginal and Torres Strait Islander women. Specifically, the two distinct aims were to:

(i) evaluate the feasibility of a centre-based women’s Aboriginal and Torres Strait Islander CR program in a non-Indigenous health setting.
(ii) investigate a combination of strategies to improve cultural safety in a non-Indigenous healthcare setting.

## Methods

### Design

The mixed methods cohort feasibility study was conducted from February to December 2018 at a University Health Clinic.

### Program design

The CR program draws on the idea of restorative healthcare, framed using restorative practices defined as “a philosophy, in action, that places respectful relationships at the heart of every interaction” [30]. This relational approach is grounded in beliefs about the equality, dignity and potential of all people and about the just structures and systems that enable people to thrive and succeed together [30]. That is, hearing vulnerable voices, acknowledging past harms, recognising and respecting culture, and applying ideas of cultural and academic reciprocity to enable dignity and respect [31]. The CR program was guided by the University Elder-in-residence and the Aboriginal and Torres Strait Islander community in a co-design model as a strategy to recognise and reduce institutional racism, a significant contributor to poor healthcare outcomes for Aboriginal and Torres Strait Islander people [32, 33]. Aboriginal and Torres Strait Islander people, including a Ngunnawal Elder, provided leadership in the research team, project reference group and an AHW was a member of the CR team. The program was developed and implemented on Ngunnawal Country and was gifted a Ngunnawal name by the United Ngunnawal Elders Council, ‘*Yeddung Gaua*r’, meaning ‘Good Heart’.

The evidence-based enablers incorporated into this program included an AHW, provision of transport assistance, and exercise and education sessions delivered in a gender specific, culturally safe, structured, yet flexible CR program in a non-hospital setting [16–19].

Environmental changes to support cultural safety included visual displays representing Aboriginal culture (Aboriginal art work, Aboriginal and Torres Strait Islander flags, map of Indigenous Australia, Aboriginal and Torres Strait Islander health posters) within the University Health Clinics [34]. Aboriginal and Torres Strait Islander resources to support cardiac risk factor modification and management were distributed to all participants [35]. Additionally, all health professionals and administrative staff involved in the program attended a half-day cultural awareness training program, approved by the University Elder-in-residence, prior to commencement of the program. The cultural awareness training program was provided by a trained Aboriginal facilitator from a local Aboriginal and Torres Strait Islander Educational Centre of Excellence. The facilitator guided health professional and administrative staff to build an understanding of Aboriginal or Torres Strait Islander identities and cultures; develop awareness of contemporary Aboriginal or Torres Strait Islander communities, including urban, rural and remote; and develop and apply cross-cultural communication skills when working with Aboriginal and Torres Strait Islander communities and people.

The CR program used a multidisciplinary team approach, including exercise physiologists, physiotherapists, dietitians, psychologists, pharmacists, supervised university health students and an AHW. The CR program was a 6-week rolling program, conducted once a week for 18-weeks (June-October 2018), consisting of 1-h exercise and 30-min education sessions. Attendance was flexible and participants were not required to attend 6-consecutive weeks, however, participants were encouraged to complete 6 sessions or more during the 18-week period. Participants were re-assessed after every sixth session attended and continued on in the program following this assessment for the remainder of the 18-weeks, if desired. The main aim of the individually tailored exercise sessions was to increase cardiovascular fitness, involving exercise at a moderate intensity [10]. The education sessions covered cardiac anatomy and physiology; risk factor modification and management; stress, anxiety and depression; medications; benefits of physical activity and nutrition advice [10]. The health professionals involved in the education sessions developed and delivered the relevant content, using a Yarning methodology. A maximum of two students attended the exercise and education sessions and were encouraged to lead these sessions under the supervision of the health professionals, where appropriate [36].

### Participants

Female Aboriginal and Torres Strait Islander adults (≥ 18 years old) were eligible for inclusion in the CR program. Participants were included if they had stable heart disease and were receiving optimal medical treatment + / − revascularisation, had experienced a heart attack, or if they were at medium-to-high risk of developing heart disease according to the Absolute Risk Calculator [37, 38]. If participants did have children < 18 years old, they were able to attend the program with their parent/carer.

### Recruitment

The program was advertised in local media (radio, print) and on the internet (online articles, Aboriginal network) from April to September 2018. Participants could self-refer or be referred by a health professional. Additionally, members of the research team and the University Elder-in-residence contacted key stakeholders such as the local AMS, Aboriginal and Torres Strait Islander health services, general medical practices, Aboriginal community groups and local hospitals. The research team also attended local Aboriginal and Torres Strait Islander events to promote the program. All potential participants were approached by the AHW initially to assess eligibility, interest and ability to commit to participating in the rolling 6-week program.

### Outcome measures

Initial assessments and collection of baseline measurements, including obtaining written consent, for all participants were conducted one week prior to the commencement of the program. Assessments were repeated every sixth session attended. Demographic and clinical information was recorded to describe the sample such as age, education level, relationship and employment status, health conditions and medication use.

The main feasibility outcome measures were participant recruitment rates, adherence to the program (number of exercise and education sessions attended), response rates to questionnaires and qualitative data on the barriers and enablers of the CR program [39]. Any adverse events experienced during the program were also recorded. Other outcome measures collected to assist with the estimation of sample size for a larger study included body mass index (BMI; kg/m^2^); waist and the hip circumference; resting blood pressure; random point-of-care lipid and glucose measurements [40]; exercise capacity (six-minute walking test, 6MWT [41]); health-related quality-of-life (MacNew Heart Disease Health-related quality-of-life Questionnaire, HRQoL [42]); and anxiety and depression (Hospital Anxiety and Depression Scale [43]).

### Cultural awareness

Assessment of health professionals’ cultural awareness pre- and post-program was conducted via a de-identified questionnaire (Additional file 1). Questionnaires were matched by health professionals providing details such as their month of birth, favourite colour and mother's month and year of birth. All staff at the University Health Clinics, both health professional and administration staff, were invited to complete the questionnaire prior to cultural awareness training and following the 18-week CR program. The questionnaire was based on a self-administered survey designed and utilised by Mooney et al. [44] to assess health professionals’ cultural awareness. It includes six questions regarding the health professionals’ perceptions of Aboriginal people; three questions on exploring health professionals’ familiarity or friendships with Aboriginal people; five statements assessing health professionals’ attitudes towards Aboriginal people; and two questions examining health professionals’ knowledge of contemporary Aboriginal health outcomes [44].

### Qualitative data

On completion of the program a yarning circle (unstructured group interview) was led by the University Elder-in-residence with participants to discuss barriers and enablers of the CR program (Additional file 2) [36]. Additionally, semi-structured telephone interviews were conducted with potential participants that did not attend the program to explore barriers and enablers to attendance (Additional file 3).

Health professionals involved in the program were invited to take part in semi-structured interviews, along with general medical practitioners, Aboriginal and Torres Strait Islander health services and cardiac rehabilitation coordinators who had referred participants to the program, to better understand barriers and enablers to attendance (Additional file 3). Alternatively, participating health professionals provided anonymous feedback using a brief online survey relating to the above issues.

### Data analysis

As this is a feasibility study a formal sample size calculation was not completed [39]. The aim was to recruit 20 participants. All participants who completed the baseline assessment and attended at least one session within the 18-week CR program were included in the analysis. For the quantitative data, descriptive analyses were completed. For the health professionals’ responses to the cultural awareness questionnaire, the Wilcoxon signed-rank test and McNemar test was used. A *p* < 0.05 was considered statistically significant. All data were analysed using SPSS version 26.

For the qualitative data, members of the research team (CB, KS, NF) independently analysed and synthesised data from the yarning group and interview transcripts and health professional online survey responses using Charmaz’s constant comparative approach, reading the transcripts multiple times [45]. Emerging themes and categories were discussed and recorded within the team until consensus was reached [46]. These methods ensured credibility, descriptive validity and minimised subjectivity for data analysis [47–49]. Member checking was not completed with participants.

## Results

### Feasibility measures, demographic and clinical descriptives

Eight referrals were received for the program. Three women were unable to attend the program due to work-time commitments and one did not attend her initial assessment. Four participants attended the program. All participants were Aboriginal females, aged between 24 and 68 years (Table 1). Two of the participants were mother and daughter. Half of the women were tertiary educated, diagnosed with heart disease or type 2 diabetes and all had at least one other chronic disease such as osteoarthritis or chronic lung disease. Most of the participants did not have a partner, were non-smokers and were not on blood pressure or cholesterol medications, although most were taking at least one medication for their heart. As this was a rolling program, participants joined the program at varying time points and therefore had various numbers of weekly sessions available, ranging from 5 to 17 sessions (median 6.5). Adherence to the weekly sessions offered ranged from 65 to 100% (median 83%; P1 80%, P2 65%, P3 86%, P4 100%).

**Table 1:** Characteristics of participants

Characteristic	Baseline (n = 4)
Age (yr), median (IQR)	41 (26–64)
Aboriginal heritage, number yes (%)	4 (100)
Relationship status, number no partner (%)	3 (75)
Education level achieved, number Tertiary (%)	2 (50)
Employment, number part-time (%)	2 (50)
Current smoker, number no (%)	3 (75)
Diagnosed with heart disease, number yes (%)	2 (50)
Diagnosed with type 2 diabetes, number yes (%)	2 (50)
Diagnosed with other chronic disease, number ≥ 1 (%)	4 (100)
Blood pressure medication, number no (%)	3 (75)
Cholesterol medication, number no (%)	3 (75)
Other heart medications, number ≥ 1 (%)	3 (75)

All participants completed baseline assessments, and only three completed the 6th session assessment, with none completing the 12th and 18th session assessments within the 18-week period (Table 2). At baseline, the average body mass index and waist circumference was above the recommended range. Blood pressure, lipid and blood glucose levels were within the normal range and participants reported they had a good health-related quality of life and low levels of anxiety and depression. No adverse events were experienced by any of the participants during the program.

**Table 2:** Comparison of participant baseline and final measures

Outcome	Baseline (n = 4)	Final^#^ (n = 3)
Weight (kg), median (IQR)	106 (78–140)	92 (77–106)
Body mass index (kg/m^2^), median (IQR)	40 (29–54)	35 (29–40)
Waist circumference (cm), median (IQR)	115 (92–133)	108 (88–115)
Waist-to-hip ratio, median (IQR)	0.88 (0.87–0.89)	0.87 (0.84–0.88)
Systolic blood pressure (mmHg), median (IQR)	117 (105–127)	110 (105–118)
Diastolic blood pressure (mmHg), median (IQR)	76 (67–79)	70 (70–73)
6-min walk test distance (m), median (IQR)	518 (290–578)	386 (246–525)
MacNew Global (*1*–*7*), median (IQR)	5.6 (5.3–6.3)	5.8 (5.7–6.0)
MacNew Physical (*1*–*7*), median (IQR)	5.2 (4.9–6.2)	6.1 (5.4–6.4)
MacNew Social (*1*–*7*), median (IQR)	6.0 (5.7–6.5)	6.2 (6.2–6.4)
MacNew Emotional (*1*–*7*), median (IQR)	5.8 (5.5–6.5)	5.7 (5.7–6.0)
HADS-Anxiety (*0*–*21*), median (IQR)	4.5 (2–7.5)	6 (3–7)
HADS-Depression (*0*–*21*), median (IQR)	3 (1.0–7.0)	2 (1.5–2.5)
Total cholesterol (mmol/L), median (IQR)	3.3 (2.9–5.1)	3.6 (3.4–3.7)
Triglycerides (mmol/L), median (IQR)	1.6 (1.1–2.2)	1.4 (1.1–2.2)
Low-density lipoprotein (mmol/L), median (IQR)	1.6 (1.6–2.2)	1.7 (1.5–2.0)
High-density lipoprotein (mmol/*L*), median (IQR)	0.88 (0.71–1.1)	0.88 (0.83–1.0)
Blood glucose level (mmol/L), median (IQR)	5.3 (5.0–5.6)	5.1 (4.7–6.2)

^#^The last cardiac rehabilitation session attended by the participant; MacNew, MacNew Heart Disease Health-related quality-of-life Questionnaire (1 = low HRQoL, 7 = high HRQoL); HADS, Hospital Anxiety and Depression Scale (higher scores indicating high anxiety and depression)

**Fig. 1:**
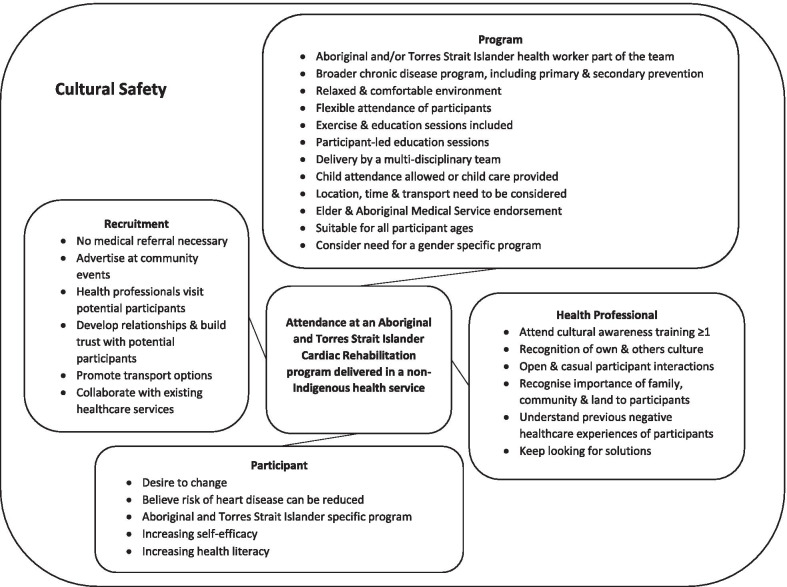
Summary of enablers for participant attendance at an Aboriginal and Torres Strait Islander Cardiac Rehabilitation program delivered in a non-Indigenous health service

At the end of the program, three of the participants took part in the yarning circle. Semi-structured interviews were conducted with one non-attendee (an Aboriginal woman that worked part-time and was at risk of developing heart disease), the AHW, four health professionals involved in the program, and four referrers. Most of the semi-structured interviews were conducted face-to-face but some were conducted via telephone. Themes identified for recruitment, program design, participant outcomes, and cultural safety and learning are outlined below. A summary of program enablers is provided in Fig. 1, with recruitment, the program and health professionals all requiring cultural safety to enable participants’ attendance.

### Community trust and time are essential for recruitment of participants

Referrers and the AHW said personal motivation to attend such programs was an issue for potential participants. They suggested there were a number of challenges Aboriginal women needed to overcome, with the desire to change their behaviour being one of them as well as recognising that their risk of heart disease could be changed.

‘I had three women who I spoke to about it and all three said, this is my destiny anyway and I can’t really change it. Everyone else in my family has died from heart disease. That’s what’s just going to happen to me.’ (Referrer, female, R2)

Both health professionals and referrers agreed it takes time to build trust with the community and the short program period (18-weeks) made this difficult. To further build trust, it was suggested recruitment should happen on the ground level, going to potential participants and building these relationships, with a large community focus. It was recommended that health professionals should build relationships with Aboriginal organisations, reaching beyond general practitioners and Aboriginal health services.

*‘really go and speak to them…actually talk to the clientele themselves…getting people comfortable with us, and then you know say, ‘oh well, maybe next week come over’’* (AHW, female, I1)

To encourage referrals, referrers needed to be aware of options for transport and access to the health centre. Potential barriers to referrals were the referrers and the AHW felt the program was in competition with other services, including the AMS, and members of the community may not have wanted to attend the program once they knew the identity of the AHW.

### Program design appeared to contribute to program acceptability and adherence

Health professionals felt this program was different to a heart health intervention, as it was more general covering other chronic diseases, such as diabetes. Although, both participants and referrers recognised that the program targeted both the primary and secondary prevention of cardiovascular disease.

*‘Yeah, it’s definitely the preventative as well, because we know black fellas are more likely to get chronic heart disease and diabetes and all of these, so we should be really getting in there early before all of that unravels.’* (Participant, female, P1)

Health professionals and participants felt that the program provided a relaxed environment, where you could talk more casually or yarn, with the discussions and education sessions being led by the participants.

*‘accommodating what they wanted, rather than us trotting in there and saying, “Well today we’re going to talk about this,” they pretty much drove the information they wanted.’* (Health professional, female, HP4)

The education sessions were generally on heart health but were broader and included discussions about family members, and health professionals felt they were increasing the health literacy of not only the participant but also family members.

Some health professionals liked the rolling nature of the program, while others did not and did not like repeating the same or similar education session to some of the same participants if they attended more than 6 sessions, with no planned discharge dates. In contrast, participants generally liked the rolling nature of the program. Some health professionals also found it frustrating when sessions needed to be cancelled at late notice due to no participants attending on that day.

*‘I was sort of here in my own time if you like, and that was, you know it was frustrating at the time, but I see that it’s part of the way these things work.’* (Health professional, male HP1)

Referrers liked that the program was delivered by a multidisciplinary team and involved exercise and education sessions. Participants and health professionals found the exercise sessions to be fun, interactive, suitable for younger participants, delivered in a comfortable environment and participants felt like they were respected. Participants also appreciated that their children (< 18 years old) were able to attend the program and were included.

Despite not attending the program, potential participants felt that the length of the program and the inclusion of exercise and education sessions was viewed as attractive, with the sessions being delivered during work-time being the major barrier to attendance, which the health professionals agreed with. Some referrers reported that potential participants felt there were too many sessions.

Both participants and referrers liked the flexible nature of the program. The health professionals and referrers liked that the program was free. Health professionals felt they needed to consider the cost to participants more than for other patient groups, for example, food preparation costs for simple meals to be made at home. Participants, health professionals and referrers all agreed that providing transport was an enabler.

Accessibility and location of the program were important factors, mentioned by the participants, AHW, referrers and health professionals. Some of the health professionals felt that delivering the program from a health facility environment may have been a barrier and some thought the program should have been offered in a different geographical location.

### The program design appeared to result in positive physical and mental health outcomes

Participants enjoyed implementing and maintaining the exercise and diet recommendations. There were positive outcomes, such as, being able to walk longer distances with less effort and cooking healthier foods at home, improving their diet.

*‘Before she started the program other people would do her shopping…[now] one of the ladies would do her shopping on her own…You know, walking around the supermarket, and then stopping, having a rest, getting up, going, that’s an achievement.’* (AHW, female, I1)

*‘I’ve really enjoyed coming to the program, I’ve learned a variety of things. How to maintain doing exercise with healthy recipes for cooking and stuff like that.’* (Participant, female, P2)

One of the referrers found that a participant had lost weight which had not been achieved with multiple previous visits to their service.

*‘she’s never lost any weight with us so we knew that wasn’t going to happen quickly…So you’ve obviously stimulated her somehow to get her moving. The weight loss, the weight loss is something that doesn’t happen easy.’* (Referrer, female, R3)

The AHW and health professionals reported participants felt empowered, they’d achieved something and it increased their personal motivation or self-efficacy. Participants, health professionals and the AHW all felt that the participants’ health literacy had increased and they were feeling more confident to question health professionals and seek advice on their health. Participants also enjoyed educating other family members about what they had learnt in the program.

### Cultural awareness training, cultural considerations and working with Aboriginal people improved cultural learning and the perception of a culturally safe space

Eighteen health professionals and administrative staff completed the cultural awareness questionnaire at baseline, before the cultural awareness training, and eleven at 18-weeks (Table 3). Nine baseline and 18-week responses were able to be matched. The majority of health professionals and administrative staff at baseline were female (n = 14/18, 78%) and between 35 and 44 years (n = 10/18, 56%), with a similar distribution found at 18-weeks. Following the cultural awareness training and involvement in the program, there was a statistically significant change in health professionals’ perception that social policies in the 1950s and 1960s helped to ‘improve’ Aboriginal people, with increasing disagreement (*p* = 0.04). No other significant changes were found. On average, participants agreed that the rate of Aboriginal people developing diabetes and presenting to hospital with heart disease was approximately 3–4 times more than non-Aboriginal people.

**Table 3:** Health professionals’ responses to the cultural awareness questionnaire at baseline and 18-weeks

Statements	Baseline (n = 18)	18-weeks (n = 11)
*Perceptions of Aboriginal Australians (1SA-5SD),* median (IQR)		
Aboriginal people in Canberra use health services more than non-Aboriginal people	4 (3–4)	5 (4–5)
You can tell who is Aboriginal just by looking at them	5 (4–5)	5 (5–5)
Aboriginal people get too much government money	4 (3.75–5)	5 (4–5)
Aboriginal people are generally well accepted in the Canberra community	3 (2–4)	3 (2–4)
Educational opportunities are unfairly given to Aboriginal people	4 (4–5)	5 (4–5)
Social policies—especially in the 1950s and 1960s—helped to improve people	4.5 (3.75–5)	5 (5–5)*
*Familiarity or friendships with Aboriginal people,* number no (%)		
Outside of work do you know any Aboriginal people reasonably well?,	9 (50)	6 (55)
Do you have any friends who are Aboriginal?	12 (67)	4 (36)
Do you work with any Aboriginal people?	11 (61)	4 (64)
*Attitudes towards Aboriginal people (1SA-5SD),* median (IQR)		
Aboriginal clients are easy to deal with	2 (2–3)	2 (2–3)
Health staff sometimes feel threatened by Aboriginal clients	3 (2–4)	3 (2–4)
Aboriginal clients tend to have more complex problems than other clients do	2 (2–3.25)	2 (1–3)
I am apprehensive about interactions with Aboriginal people	4 (4–5)	4 (4–5)
Aboriginal health problems are largely due to changes in lifestyle and diet	3 (2–4)	4 (2–4)
*Knowledge of contemporary Aboriginal health outcomes (1 ‘a bit less’—5 ‘more than 10 times the rate’),* median (IQR)		
What is the rate of developing diabetes in Aboriginal people, compared to non-Aboriginal people	4 (3.75–4)	4 (4–5)
What is the rate of presentations with heart disease by Aboriginal people to hospital (emergency departments, inpatients), compared to non-Aboriginal people	4 (2.75–4.5)	4 (3–5)

*SA* strongly agree, *SD* strongly disagree

*p < 0.05;

### Participant and health professional interactions

Participants and health professionals felt that the program increased the knowledge, skills and confidence of the health professionals in working with Aboriginal patients. Health professionals, the AHW and participants said the AHW involvement was crucial, they were seen as a navigator of the program for the Aboriginal women.

*‘having her there was important for the participants…it was in a way bridging the gap. If she wasn’t there then the adherence to participants turning up may have been affected…I think it’s got to do with the respect or the trust that participants will have in accessing services.’* (Health professional, female, HP2)

The AHW suggested health professionals need to get to know Aboriginal people individually, to improve their interactions. Health professionals learnt to be more open, more casual and talk more freely about their family which they wouldn’t normally do in a patient interaction.

*‘I found that it was little bit different in the sense that you could be a little bit more open with them, because the more open that you actually are, the more open they are. So I found that I needed to just be in a way a little bit more casual in a way sometimes, because they’d ask questions and you’re just like, “Oh, that’s,” you know I wouldn’t normally speak about [my] family, because they are massive on family, but I would normally not speak about [my] family with clients… At first I was a bit hesitant, because obviously you know we were taught basically to keep it professional, so opening up in that sense was a little bit more vulnerable.’* (Health professional, female, HP2)

Health professionals found it took time to gain the trust of the participants and build rapport.

*‘I found the participants, it took a bit of time to actually warm up and I guess trust and build that rapport with them, and then once that developed then it was much easier to interact with them, and then for them to trust me with the program that I had recommended.’* (Health professional, female, HP2)

Health professionals said they needed to recognise their own culture and the culture of the patients, including allowing time for silence. They also found that analogies worked well, particularly connecting with the land.

### Cultural awareness training

Health professionals reported that the cultural awareness training increased their confidence with interactions with Aboriginal and Torres Strait Islander people.

*‘Yes, I think it just made me a little bit more comfortable. So not having a lot to do with Indigenous people, I just didn’t, you know it was a bit I thought, well do I say the wrong thing? But once I got to know them and realised that I didn’t have to be frightened about what I said, well you know I wasn’t… you know with any group you’re careful what you say, but I’ve certainly felt that I was, you know, able to… and once I’ve met them. So having that initial information and understanding of the background, and where, what’s influencing, you know, people’s behaviours, I thought that made it a lot more easier’* (Health professional, female, HP4)

Health professionals, referrers and the AHW recommended that attendance at cultural awareness training sessions should be repeated and not completed once only, similar to other mandatory workplace training, and the AHW suggested that cultural awareness training alone was not enough.

*‘maybe every one or two years have the opportunity to attend something like [cultural awareness training], because it’s kind of like first aid, you learn something new every time you go, no matter how much you’re like, oh, I’ve done this before.’* (Health professional, female, HP3)

### Program cultural considerations

Referrers, the AHW and participants discussed participant’s previous negative experiences with institutions and health professionals, reporting feelings of shame, guilt and judgement.

*‘OK, I’ve done the mainstream program, well I was put into it, at a point. Because I missed an appointment, because something came up, some sorry business came up. And because I missed that appointment, they just cancelled the rest of the appointments.’* (AHW, female, I1)

The flexibility of the program was greatly valued as this lessened the feeling of guilt if participants were unable to attend every week.

*‘It was a relaxed environment. It wasn’t rigid. Like I’ve said before, there’s been in mainstream, when you go there, you feel like you’re carrying guilt for being sick in the services. So here was, we could come in, if we were a bit off, they left us to do what we had to do. Didn’t question us.’* (Participant, female, P3)

*‘I mean people aren’t going to go to…like the shame and guilt, you know, we’re our own worst enemies, if we don’t turn up to something we feel guilty’* (AHW, female, I1)

Although some health professionals thought the flexibility of the program may not encourage consistency.

*‘I do think having a capacity for people to not feel like if you don’t attend for your six weeks you miss out…I think that works really well, but it also doesn’t necessarily lead to great buy-in if you know that you can just turn up at any time. So I don’t know if there’s a way to find that middle ground…’* (Health professional, female, HP3)

Health professionals and referrers felt they needed to recognise that they were working with individuals where family and community would always come first.

*‘you saw that real connectedness in just participants… that just made me realise that you’re actually working with a very connected community’* (Health professional, female, HP3)

Allowing children (< 18 years old) to attend the program was very valuable to health professionals and participants, allowing family sharing of both participants and health professionals and a personal connection.

Participants felt this was a safe and comfortable environment, with *‘black faces’*, and being Aboriginal and Torres Strait Islander specific was a positive. The program differed to a mainstream service and was not time limited.

*‘ [the] program was incredible, especially having Aboriginal women there supporting you, like [the AHW]… So even just having that space to yarn, it was good.’* (Participant, female, P1)

Some health professionals felt that a culturally safe space was provided by introducing Aboriginal and Torres Strait Islander flags and artwork but some health professionals did not notice this.

*‘I don’t think people really take that much notice of it. I think… I don’t know whether people are sort of used to seeing Aboriginal artworks around, and maybe they just don’t [notice it]… But it definitely needs to be acknowledged, that’s for sure.’* (Health professional, female, HP4)

Some referrers suggested that potential participants did not want to go to the AMS, while others felt that AMS endorsement was important. Elder guidance and endorsement of the program was viewed as very important.

*‘if they’re not promoting it, is it really for our mob?’* (Referrer, female, R2)

Referrers, participants and the AHW all agreed that cultural considerations are important, particularly taking into consideration that heart problems can occur in younger people in the Aboriginal community. The resources provided were thought to be culturally appropriate by the health professionals and referrers, although they were not tailored for all participants, particularly younger participants. Health professionals questioned whether the program needed to be gender specific, while the AHW, participants and referrers thought this was a positive for the program.

## Discussion

The Yeddung Gauar program created a culturally safe space for Aboriginal women using a combination of strategies to address non-Indigenous health professionals’ knowledge, awareness, behaviours, skills and attitudes. Participants felt that the health service environment was comfortable and safe, where they were not judged, and lessened feelings of guilt connected with previous experiences within the mainstream healthcare system. Health professionals’ knowledge, skills and confidence in working with Aboriginal people improved following attendance at the cultural awareness training and involvement in the program. However, issues such as recruitment methods need to be addressed to increase community trust and referrals to similar programs.

It is clear that cultural awareness training alone is not sufficient to improve cultural safety [24]. This is supported by Mooney et al. [44] who used a quasi-experimental design to evaluate the impact of Aboriginal cultural awareness training in a New South Wales urban health service. The authors surveyed non-Indigenous health professionals before and after a cultural awareness training half-day workshop and concluded that half-day workshops did not significantly change beliefs and attitudes towards Aboriginal people. Here we found a change in knowledge and awareness. Using a combination of cultural awareness training and involvement in the co-designed program with AHW support, there was a statistically significant change in health professionals’ perception that social policies in the 1950s and 1960s helped to improve Aboriginal people, with increasing disagreement. Non-Indigenous health professionals also reported changing their behaviours and clinical skills as a result of working in the Yeddung Gauar program, learning to be more open and less structured in their patient-health professional interactions. The health professionals developed partnerships with participants, where participants led discussions and education sessions, eliminated bias through self-reflection and built relationships with participants. Previous studies have found this is the key to reducing health inequalities for Indigenous populations [24]. Although, the value of cultural awareness training should not be dismissed as it did contribute to health professionals’ confidence to interact with Aboriginal and Torres Strait Islander people but repeated attendance was strongly recommended by the AHW, health professionals and referrers.

Themes of relationship and trust building, and unconditional positive regard towards attendees, with no judgement were strong and contributed greatly to cultural safety, from the participants’ perspective. This may have contributed to the adherence to the program, with all participants attending > 65% of the sessions available to them, higher than other rates of attendance previously reported in Aboriginal specific CR programs [17]. Health professionals need to be culturally safe and sensitised to the impact of institutional racism and white privilege as well as allow flexibility within the program to allow participants’ not to feel guilty when they could not attend. The need to embed cultural safety into our health systems has been recognised by the Australian Health Practitioner Regulation Agency, releasing a strategy in early 2020 with an objective to provide a culturally safe health workforce through consistent standards, codes and guidelines across all registered health practitioners in Australia [26]. Despite making efforts to provide a culturally safe space once participants were in the program, the AHW and referrers suggested this was also needed during the recruitment phase, otherwise recruitment to the program may continue to be low.

As part of the program non-Indigenous health professionals recognised how important family and community were to participants. Hence, non-Indigenous health professionals’ need to be cognisant that the Aboriginal definition of health (life-death-life) differs from the Western view of absence of disease. Aboriginal health means not just the physical well-being of an individual but refers to the social, emotional and cultural well-being of the whole Community in which each individual is able to achieve their full potential as a human being thereby bringing about the total well-being of their Community, with less focus on the individual [50].

The known enablers to increase participation in CR for Aboriginal and Torres Strait Islander peoples that were utilised in this program such as the inclusion of an AHW, provision of transport and a culturally safe environment, and a gender-specific flexible program reinforced the value of such factors, with all them found to be major enablers for the Yeddung Gauar program, resulting in positive changes in health outcomes [16–19]. These factors have also been found to be important to other Indigenous communities around the world [51]. However, the main barriers to program attendance were not unique to this population, such as competing work and carer commitments, and timing of the program [52].

### Restorative practice and its implication for future programs

An important learning from this research was that although included as an investigator in the study, the leadership of an Aboriginal Traditional Land-owner and Elder could have been utilised more effectively in the co-design, management and leadership within the program. A barrier to success was noted to be the traditional colonised and hierarchical approach to research and health systems that does not take into account the cultural connection to Country, and respect and leadership power that Traditional Land-owner Elders hold in their local community. It is critical to be aware of Aboriginal protocols so as not to offend Aboriginal people. It is vital not to diminish the role of Traditional Owners in understanding the diversity that exists across each of the Aboriginal nations and respecting the difference between knowledges held in each First nation. Non-Indigenous researchers must practice cultural humility to have success and be alert to the reality that Aboriginal and/or Torres Strait Islander Health Workers who are not from the same Country need support and guidance from Elders who are Traditional Owners to successfully engage with the Aboriginal community they are working within. Non-Indigenous researchers and health workers need to respect the Elders guidance and leadership for trust to grow. Elders are leaders who are respected and followed by their community and will actively encourage participation when actively engaged. Using approaches that ensure cultural sensitivity, respect and voice of Traditional Land-owner Elders to guide the work and ensure all team members are respected will enhance future programs. In the gifted words of the United Ngunnawal Elders who supported this project, “In this Journey we strive for unity. We do this by empowering people, creating confidence, self-esteem and room for difference, so we can work and laugh together, moving forward all the while” [53].

### Strengths, limitations and future directions

Three of the research team are First Australians (RB, PC, CB). Researchers from physiotherapy and nursing with experience in Indigenous health, cardiac rehabilitation and restorative practice have also been involved in this research. An important strength of this study is the deliberate privileging of Aboriginal and Torres Strait Islander voices at all levels of the research strategy, applying restorative healthcare practice principles, an ontologically appropriate strength-based approach for this study with foundations in First Nations practice [54, 55]. The findings from this mixed-methods study provides authentic and credible qualitative information from participants and providers, to guide and inform the development and implementation of future studies and clinical practice. As this was a feasibility study, the sample size was very small, and the quantitative results should be interpreted with caution [39]. The Aboriginal and Torres Strait Islander population in Canberra is also relatively small, which may have limited recruitment [56]. Further research is indicated with larger sample sizes, an extended program and longer follow-up to determine the efficacy of the program. Future research should focus on strategies to increase the recruitment of participants to such programs, working in partnership with and being guided by Traditional Land Owner Aboriginal and Torres Strait Islander Elders and communities, developing a culturally safe and acceptable environment between healthcare providers and the community before entering the program.

## Conclusion

The Yeddung Gauar cardiac rehabilitation program enabled female Aboriginal and Torres Strait Islander participants to attend a gender-specific evidence-based cardiac rehabilitation program, for the primary and secondary prevention of heart disease. This program was effectively delivered in a non-Indigenous health service by engaging in strategies that improved health workforce cultural safety and resulted in positive health outcomes. Future programs in non-Indigenous health settings should employ similar strategies and cultural learnings to increase cultural safety, recognising the evidence-based enablers and barriers for cardiac rehabilitation attendance for both Indigenous and non-Indigenous Australians.

## Supplementary Information

**Additional file1:** Health professionals cultural awareness questionnaire.

**Additional file2:** Yarning circle topic guide.

**Additional file3:** Semi-structured interview topic guide.

## Data Availability

The data sets used and analysed during the current study are available from the corresponding author on reasonable request.

## Abbreviations

6MWT: 6-min walk test
AHW: Aboriginal and/or Torres Strait Islander health worker
AMS: Aboriginal Medical Service
BMI: Body mass index
CR: Cardiac rehabilitation
CVD: Cardiovascular disease
HRQoL: Health-related quality-of-life
