# Supplementary material for: An Aboriginal and Torres Strait Islander Cardiac Rehabilitation program delivered in a non-Indigenous health service (Yeddung Gauar): a mixed methods feasibility study
Source: BMC Cardiovasc Disord. 2021 May 1;21:222. doi: 10.1186/s12872-021-02016-3 (PMC8088627; doi:10.1186/s12872-021-02016-3)
Supplement: Supplementary file 1 — Additional file1: Health professionals cultural awareness questionnaire. [file 12872_2021_2016_MOESM1_ESM.docx]

**
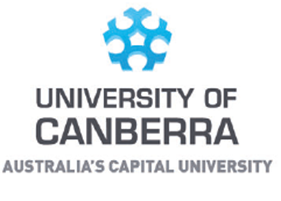
**

This survey asks questions about your perceptions, familiarity and friendships, attitudes and knowledge of Australian Aboriginal and Torres Strait Islanders and the health issues affecting them. Thank you for completing these questions.

The questions below are designed to help us to match the de-identified questionnaires pre and post-program.

| **Month of Birth** |  | **Today’s Date** |  |
| --- | --- | --- | --- |
| **Month/Year of Mother’s Birth** |  | | |
| **Favourite colour** |  | | |

**1. To what extent do you agree or disagree with the following statements: *(please circle only one for each statement)***

|  | **Strongly**  **Agree** | **Agree** | **Not**  **Sure** | **Disagree** | **Strongly**  **Disagree** |
| --- | --- | --- | --- | --- | --- |
| a. Aboriginal people in Canberra use health services more than non-Aboriginal people | 1 | 2 | 3 | 4 | 5 |
| b. You can tell who is Aboriginal just by looking at them | 1 | 2 | 3 | 4 | 5 |
| c. Aboriginal people get too much government money | 1 | 2 | 3 | 4 | 5 |
| d. Aboriginal people are generally well accepted in the Canberra community | 1 | 2 | 3 | 4 | 5 |
| e. Educational opportunities are unfairly given to Aboriginal people | 1 | 2 | 3 | 4 | 5 |
| f. Social policies – especially in the 1950s and 1960s – helped to improve people | 1 | 2 | 3 | 4 | 5 |

**2. Please circle ‘yes’ or ‘no’ for the following questions.**

a. Outside of work do you know any Aboriginal people reasonably well ? Yes No

b. Do you have any friends who are Aboriginal? Yes No

c. Do you work with any Aboriginal people? Yes No

**3. To what extent do you agree or disagree with the following statements: *(please circle only one for each statement)***

|  | **Strongly**  **Agree** | **Agree** | **Not**  **Sure** | **Disagree** | **Strongly**  **Disagree** |
| --- | --- | --- | --- | --- | --- |
| a. Aboriginal clients are easy to deal with | 1 | 2 | 3 | 4 | 5 |
| b. Health staff sometimes feel threatened by Aboriginal clients | 1 | 2 | 3 | 4 | 5 |
| c. Aboriginal clients tend to have more complex problems than other clients do | 1 | 2 | 3 | 4 | 5 |
| d. I am apprehensive about interactions with Aboriginal people | 1 | 2 | 3 | 4 | 5 |
| e. Aboriginal health problems are largely due to changes in lifestyle and diet | 1 | 2 | 3 | 4 | 5 |

**4. The rate of developing diabetes in Aboriginal people, compared to non-Aboriginal people, is *(please select one)***

○ a bit less

○ about the same

○ about 1.5 times the rate

○ about 3-4 times the rate

○ More than 10 times the rate

**5. The rate of presentations with heart disease by Aboriginal people to hospital (emergency departments, inpatients), compared to non-Aboriginal people, is *(please select one)***

○ a bit less

○ about the same

○ about 1.5 times the rate

○ about 3-4 times the rate

○ More than 10 times the rate

○ not sure/don’t know

**5. Some general questions about you:**

c. Your gender: ○ Female ○ Male

d. Your age in years: ○ <35 ○ 35-44 ○ 45-54 ○ >54

**Thank you for taking the time to complete this survey ☺**
