# Supplementary material for: An Aboriginal and Torres Strait Islander Cardiac Rehabilitation program delivered in a non-Indigenous health service (Yeddung Gauar): a mixed methods feasibility study
Source: BMC Cardiovasc Disord. 2021 May 1;21:222. doi: 10.1186/s12872-021-02016-3 (PMC8088627; doi:10.1186/s12872-021-02016-3)
Supplement: Supplementary file 2 — Additional file2: Yarning circle topic guide. [file 12872_2021_2016_MOESM2_ESM.docx]

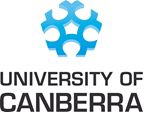


*‘Yeddung Gauar’: Feasibility of a women’s Aboriginal and Torres Strait Islander Cardiac Rehabilitation program delivered in a non-Indigenous health service.*

Topic Guide – Program Evaluation participants

(Used to guide participant Yarning Circle)

| Topic |
| --- |
| **Introduction**   - General purpose of the session   eg: “*talk about your experience with participating in Yeddung Gauar”* |
| **Participants impressions about the content and delivery of the exercise and education sessions**   - Consider the structure of the program (eg: rolling program, once a week, hour of exercise, 30 mins for education, 12-2pm, aim for participants to attend at least 6 sessions which did not have to be consecutively, gender specific, transport organised, ALO involved), resources provided (eg: heart foundation resources for Aboriginal and Torres Strait Islanders, including ‘My Heart, My Life’, and food provided at each session) |
| **Resources: their content and use**   - See above |
| **Barriers and facilitators to the program**   - See above |
| **Relationship development between the health professionals and participants**   - Specifically ask questions around cultural safety eg: did you feel culturally safe attending the program? - Did the space feel culturally welcoming eg: did you notice the Aboriginal and Torres Strait Islander flags at Reception and the Aboriginal artwork? |
| **Future suggested strategies** |
| **Close**   - Summarise - Further comments |

Reference: 1. McRae, M., et al., *Evaluation of a pharmacist-led, medicines education program for Aboriginal Health Workers.* Rural And Remote Health, 2008. **8**(4): p. 946-946.
