# Supplementary material for: An Aboriginal and Torres Strait Islander Cardiac Rehabilitation program delivered in a non-Indigenous health service (Yeddung Gauar): a mixed methods feasibility study
Source: BMC Cardiovasc Disord. 2021 May 1;21:222. doi: 10.1186/s12872-021-02016-3 (PMC8088627; doi:10.1186/s12872-021-02016-3)
Supplement: Supplementary file 3 — Additional file3: Semi-structured interview topic guide. [file 12872_2021_2016_MOESM3_ESM.docx]

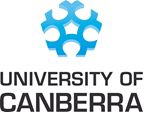


*‘Yeddung Gauar’: Feasibility of an Aboriginal and Torres Strait Islander Cardiac Rehabilitation program delivered in a non-Indigenous health service.*

Topic Guide – Program Evaluation participants/health professionals

(Used to guide participant unstructured interviews, health professional/referrer interviews, health professional anonymous online survey)

| Topic |
| --- |
| **Introduction**   - General purpose of the session   eg: “*talk about your experience with participating in/conducting Heart Time”* |
| **Participants/health professionals impressions about the content and delivery of the exercise and education sessions** |
| **Resources: their content and use** |
| **Barriers and facilitators to the program** |
| **Relationship development between the health professionals and participants** |
| **Future suggested strategies** |
| **Close**   - Summarise - Further comments |

Reference:

[[1](#_ENREF_1)]1. McRae, M., et al., *Evaluation of a pharmacist-led, medicines education program for Aboriginal Health Workers.* Rural And Remote Health, 2008. **8**(4): p. 946-946.
